# Prof. Hamilton

**Published:** 1866-06

**Authors:** 


PROF. HAMILTON is preparing a new edition of his work on Fractures and Dislocations.

